# Neuroprotective effects of bee venom phospholipase A2 in the 3xTg AD mouse model of Alzheimer’s disease

**DOI:** 10.1186/s12974-016-0476-z

**Published:** 2016-01-16

**Authors:** Minsook Ye, Hwan-Suck Chung, Chanju Lee, Moon Sik Yoon, A. Ram Yu, Jin Su Kim, Deok-Sang Hwang, Insop Shim, Hyunsu Bae

**Affiliations:** Department of Physiology, College of Korean Medicine, Kyung Hee University, #1 Hoegi-dong, Dongdaemoon-ku, Seoul, 130-701 Republic of Korea; Korean Medicine (KM)-Application Center, Korea Institute of Oriental Medicine (KIOM), 70, Cheomdan-ro, Dong-gu, Daegu, 41062 Republic of Korea; Molecular Imaging Research Center, Korea Institute of Radiological and Medical Sciences, University of Science and Technology, #215-4 Gongneug-dong, Nowon-ku, Seoul, 139-241 Republic of Korea; Department of Obstetrics and Gynecology, College of Korean Medicine, Kyung Hee University, #1 Hoegi-dong, Dongdaemoon-ku, Seoul, 130-701 Republic of Korea; Acupuncture and Meridian Science Research Center, College of Korean Medical Science Graduate School, Kyung Hee University, #1 Hoegi-dong, Dongdaemoon-ku, Seoul, 130-701 Republic of Korea

**Keywords:** Alzheimer’s disease, Regulatory T cells, Bee venom phospholipase A2, Microglia, Neuroinflammation, PET

## Abstract

**Background:**

Alzheimer’s disease (AD) is a severe neuroinflammatory disease. CD4^+^Foxp3^+^ regulatory T cells (Tregs) modulate various inflammatory diseases via suppressing Th cell activation. There are increasing evidences that Tregs have beneficial roles in neurodegenerative diseases. Previously, we found the population of Treg cells was significantly increased by bee venom phospholipase A2 (bvPLA2) treatment in vivo and in vitro.

**Methods:**

To examine the effects of bvPLA2 on AD, bvPLA2 was administered to 3xTg-AD mice, mouse model of Alzheimer’s disease. The levels of amyloid beta (Aβ) deposits in the hippocampus, glucose metabolism in the brain, microglia activation, and CD4^+^ T cell infiltration were analyzed to evaluate the neuroprotective effect of bvPLA2.

**Results:**

bvPLA2 treatment significantly enhanced the cognitive function of the 3xTg-AD mice and increased glucose metabolism, as assessed with 18F-2 fluoro-2-deoxy-D-glucose ([F-18] FDG) positron emission tomography (PET). The levels of Aβ deposits in the hippocampus were dramatically decreased by bvPLA2 treatment. This neuroprotective effect of bvPLA2 was associated with microglial deactivation and reduction in CD4^+^ T cell infiltration. Interestingly, the neuroprotective effects of bvPLA2 were abolished in Treg-depleted mice.

**Conclusions:**

The present studies strongly suggest that the increase of Treg population by bvPLA2 treatment might inhibit progression of AD in the 3xTg AD mice.

## Background

Alzheimer’s disease (AD) is a degenerative disease of the central nervous system. AD is pathologically characterized by extracellular senile plaques, intracellular neurofibrillary tangles, and a reduction of neurons in the cerebral cortex and hippocampus. The clinical features of AD are loss of memory and cognitive and behavioral disorders [1]. AD is one of the most common types of dementia, but its etiology and pathogenesis remain unclear. At present, various pathological processes are known to be involved in AD. However, the amyloid cascade hypothesis and the amyloid beta (Aβ) toxicity hypothesis have dominated research for decades. And the deposition of Aβ peptide in the brain is considered a central event in AD [2, 3]. Although the etiology of AD is unknown, many evidences suggest that inflammatory responses play a crucial role in its pathogenesis [4, 5]. Inflammatory processes that are marked by activated microglia and astrocytes, some of which co-localize to plaques and tangles have long been hypothesized to contribute to AD pathogenesis [6]. Activated microglia have been found in the brains of AD patients with dementia and or patients with mild cognitive impairment [7, 8]. Microglia, the resident immune cells of the central nervous system, are activated to phagocytize Aβ [9], but the impaired clearance or reuptake of Aβ results in the release of inflammatory cytokines that cause neuroinflammation [10]. Therefore, microglial activation might contribute to the deposition of Aβ and the progression of AD [11].

At present, there is absolutely no significant treatment for AD. The current mainstays of treatment for cognitive loss related to AD are muscarinic or nicotinic receptor ligands and acetylcholinesterase (AChE) inhibitors [12]. However, these drugs have undesirable side effects that include diarrhea, nausea, vomiting, muscle cramps, sedation, and bradycardia [13]. Despite many years of intensive research, the mechanisms that trigger the disease remain uncertain, and there is currently no promising treatment [14]. Accordingly, the identification of effective and safe treatments with clear pharmacological mechanisms is urgently needed.

In the present study, we used a triple-transgenic model (3xTg-AD mouse) that is unique among previous models because it expresses three dementia-related transgenes, i.e., namely APP_Swe_, PS1_M146V_, and tau_P301L_ and exhibits a clear age-dependent onset of AD neuropathology [15]. Extracellular Aβ deposits have been shown to be apparent at 6 months and are predominantly located in the hippocampus and frontal cortex [16, 17].

Phospholipase A2 (PLA2) is an enzyme that catalyzes the hydrolysis of the sn-2 fatty acyl bond of membrane phospholipids to produce free fatty acids and lysophospholipids [18, 19]. PLA2 is present in various species and has been categorized into three broad classes based on cellular distribution: a secreted or extracellular type (sPLA2), a cytosolic or intracellular type (cPLA2), and a Ca2^+^-independent PLA2. In addition to mammals, sPLA2 is present in the venom of snakes, bees, cnidarians, and scorpions. sPLA2 plays important roles in a wide range of cellular responses, including phospholipid metabolism, signal transduction, and the initiation and regulation of inflammatory and immune responses [20–23].

It has previously been reported that bee venom possesses therapeutic effects in the 1-methyl-4-phenyl-1,2,3,6-tetrahydropyridine (MPTP)-induced Parkinson’s disease model that are mediated via the modulating of Treg populations. And we also reported that bvPLA2 treatment induce Treg population in vivo and in vitro [24]. In the present study, we examined the effects of PLA2 from bee venom (bvPLA2) on the learning and memory abilities of 3xTg-AD mice using the Morris water maze. We further sought to determine whether bvPLA2 decreases Aβ in the 3xTg mouse model of AD. We also demonstrated that the neuroprotective effects of bvPLA2 were associated with the suppression of microglial activation via the modulation of peripheral immune tolerance by Tregs. Furthermore, we examined brain activity using positron emission tomography (PET) with 18F-2 fluoro-2-deoxy-D-glucose ([F-18] FDG), which enables the autoradiographic assessment of radiolabeled 2-deoxyglucose uptake.

## Methods

### Animals and treatment

bvPLA2 (Sigma-Aldrich, St. Louis, MO, USA) was dissolved in sterile phosphate-buffered saline (PBS) and kept at −20 °C until use. The 3xTg AD mice harboring a mutant APP (KM670/671NL), which is a human mutant PS1 (M146V) knock-in, and tau (P301L) transgenes (B6;129-*Psen1*^*tm1Mpm*^ Tg(APPSwe, tauP301L)1Lfa/J) [15] were purchased from Jackson Laboratory (Bar Harbor, ME, USA). Non-transgenic littermates were used as wild type (WT) controls. The animal procedures were approved by the University of Kyung Hee Institutional Animal Care and Usage Committee (KHUASP(SE)-13-015) and were in accordance with the Guide for the Care and Use of Laboratory Animals of the National Institutes of Health. All animals were maintained in a pathogen-free environment on a 12-h light/dark cycle and had access to food and water ad libitum. The mice were randomly assigned to five groups as follows: (1) a PBS-treated WT group (WT, *n* = 6); (2) a PBS-treated 3xTg group (3xTg, *n* = 5); (3) a 0.6 mg/kg donepezil-treated 3xTg group (3xTg+donepezil, *n* = 6); (4) a 0.2 mg/kg bvPLA2-treated 3xTg group (3xTg+bvPLA2 0.2, *n* = 6); and (5) a 1 mg/kg bvPLA2-treated 3xTg group (3xTg+bvPLA2 1, *n* = 4). Donepezil was used as a positive control and was purchased from Sigma (Sigma, St. Louis, MO, USA). Beginning at 3 months of age, PBS, bvPLA2, or donepezil was intraperitoneally administered for 3 months once per week until they were 6 months old. For the Treg depletion, anti-mouse CD25 rat IgG (anti-CD25, clone PC61) antibodies were generated in-house from hybridomas obtained from the American Type Culture Collection (Manassas, VA, USA). The mice received doses of 1 mg/kg of anti-CD25 rat IgG or normal anti-rat IgG1 once per week for 3 months until they were 6 months old. The efficacy of the CD4^+^ CD25^+^ Foxp3^+^ Treg cell depletion was determined by flow cytometry (FACS, FACSCalibur, BD Biosciences, San Jose, CA, USA) using anti-CD25-PE, anti-CD4-FITC, and anti-Foxp3-Cy5 PE (eBiosciences).

### Morris water maze test

A modified version of the water maze procedure reported by Morris was used [25]. The water maze was a circular pool of 90 cm in diameter that was constructed of fiberglass. The pool contained water that was maintained at a temperature of 22 ± 2 °C and contained 1 kg of powdered skim milk to make the water opaque. During the water maze testing, a platform (6 cm in diameter) was fixed at 1 cm below the surface of the water at an identical location within the pool. The pool was surrounded by different extra-maze cues. Each trial was initiated at one of the different starting positions, and the swimming path of each mouse was recorded with a video camera connected to a video recorder and a tracking device (S-MART, Pan-Lab, Spain). All mice were subjected to four trials per day at intervals of 15 min for four consecutive days followed by 1 day of probe trials on the fifth day. The trials were considered to be completed when the mice found the hidden platform or the escape latency reached 60 s. For the probe trials, the platform was removed from the pool, and the mice were put in the pool for 60 s to search for the previous location of the platform. The memory retention was measured by the percentage of time spent searching for the platform in the training quadrant.

### Tissue preparation and immunohistochemical staining

The mice were transcardially perfused with saline solution with 0.5 % sodium nitrate and heparin (10 U/ml) and then fixed with 4 % paraformaldehyde (PFA) in 0.1 M phosphate buffer (PB). Each brain was dissected from the skull, post-fixed overnight in 4 % PFA at 4 °C, stored in a 30 % sucrose solution at 4 °C until it sank, and frozen-sectioned on a sliding microtome into 30-μm-thick coronal sections. All of the sections were processed for immunohistochemical (IHC) staining as described previously [26]. The brain sections were washed with PBS and incubated overnight at room temperature with primary antibody. The next day, the brain sections were washed with PBS with 0.5 % bovine serum albumin (BSA), incubated with the biotinylated secondary antibody, and processed with an avidin-biotin complex kit (Vectastain ABC kit; Vector Laboratories, Burlingame, CA, USA). The bound anti-serum was visualized by incubation with 0.05 % diaminobenzidine-HCl (DAB) and 0.003 % hydrogen peroxide in 0.1 M PB. The DAB reaction was stopped by washing the slide section with 0.1 M PB. The primary antibodies that were used were directed against Aβ (1:100; Abcam, Cambridge, MA, USA), CD11b (1:200; Serotec, Oxford, UK), and CD4 (1:200; Serotec). The tissue sections were then mounted on gelatin-coated slides and observed under a bright-field microscope (Nikon, Tokyo, Japan).

### Flow cytometric analysis

After the behavioral tests, single-cell suspensions of splenocytes were stained with fluorescently tagged CD4, CD25, and Foxp3 (eBiosciences, San Diego, CA, USA) in accordance with the manufacturer’s instructions. All FACS data were acquired using a FACSCalibur flow cytometer (BD Biosciences) and analyzed using CellQuest Pro (BD Biosciences).

### Immunohistochemistry and quantification

Coronal sections of the hippocampus, starting rostrally from anteroposterior −2.1 mm and continuing to anteroposterior −4.5 mm relative to bregma, were collected. The numbers of CD11b-positive microglia/macrophages and CD4^+^ T cells in the hippocampi stained with single-label immunohistochemistry were counted under a ×400 objective (250 μm) using a light microscope (Nikon).

### Densitometry analysis

The coronal sections of the hippocampus, starting rostrally from anteroposterior −2.1 mm and continuing to anteroposterior −4.5 mm relative to bregma, were examined at ×10 magnification using the IMAGE PRO PLUS system (version 4.0; Media Cybernetics, Silver Spring, MD, USA) on a computer attached to a light microscope (Zeiss Axioskop, Oberkochen, Germany) that was interfaced with a charge-coupled device video camera (Kodak Mega Plus model 1.4 I). To determine the density of the Aβ-immunoreactive, CD11b-positive microglia/macrophages, and CD4^+^ T cells staining in the hippocampus, a square frame of 500 × 500 μm was placed on the dorsal part of the hippocampus. A second square frame of 200 × 200 μm was placed in the region of the corpus callosum to measure the background value. To control for variations in background illumination, the average of the background density readings from the corpus callosum was subtracted from the average of the density readings from the hippocampus for each section.

### [F-18] FDG micro-PET scan

All the mice were fasted for 12–15 h before the experiments to enhance the [F-18] FDG uptake in the brain [27]. Before the [F-18] FDG injection, all mice were put on a heating pad in a cage and warmed for at least 30 min. The temperatures of the cages were maintained at 30 °C throughout the uptake period in accordance with an optimized [F-18] FDG uptake protocol [28]. [F-18] FDG (500 μCi/100 g body weight) was injected through a tail vein, and the mice were anesthetized with 2 % isoflurane in 100 % oxygen (Forane solution; ChoongWae Pharma, Seoul, Korea). For the PET imaging, a Siemens Inveon PET scanner (Siemens Medical Solutions, Malvern, PA, USA) was used throughout the study. The transverse resolution was used was <1.8 mm at the center [29, 30]. Transmission PET data were acquired for 15 min using a Co-57 point source with an energy window of 120–125 keV. One mCi of [F-18] FDG was injected. Thirty min after injection of 1 mCi of [F-18] FDG, which period allowing adequate time for tracer uptake, emission PET data during 30 min were acquired within an energy window of 350-650 keV. The emission list-mode PET data were sorted into 3D sinograms and reconstructed using three DRP methods. The pixel size of the reconstructed images was 0.15 × 0.15 × 0.79 mm^3^. Attenuation and scatter corrections were performed for all of the datasets.

### Voxel-based statistical analysis

Voxel-based statistical analysis was performed to compare the cerebral glucose metabolisms of the datasets of the different groups. The procedure used for the statistical parametric mapping (SPM) analysis of the animal PET data has previously been described [31]. Briefly, for efficient spatial normalization, only the brain region was extracted. A study-specific template was then constructed using all of the datasets. The PET data were spatially normalized onto a mouse brain template and smoothed using a Gaussian kernel. Count normalization was performed. A voxel-wise *t* test between the groups’ datasets was performed using the Statistical Parametric Mapping 5 program (*P* < 0.05, *K* > 50) (Table 1).

**Table 1:** The results of voxel-wise comparisons. The results of voxel-wise comparisons between the WT and 3xTg datasets (A), the 3xTg and 3xTg+donepezil datasets (B), and the 3xTg and 3xTg+bvPLA2 1 mg datasets (C)

Group	Brain area	Coordinates (x, y, z)	Cluster size	*Z* value
A	Diencephalon	(−1, 3, −2)	16940	2.62
B	Cortex	(0, 1, −3)	2388	1.82
C	Diencephalon	(−2, 3, 0)	3359	2.15

### Statistical analysis

Statistical comparisons for the behavioral and histochemical studies were performed with one-way ANOVAs followed by Tukey’s post hoc tests. All values are expressed as the mean ± the SEM, and Prism 5.01 software (GraphPad Software Inc., San Diego, CA, USA) for Windows was used for the statistical analyses. The significance level was set at *P* < 0.05.

## Results

### Effect of bvPLA2 treatment on the body weights of the mice

**Fig. 1:**
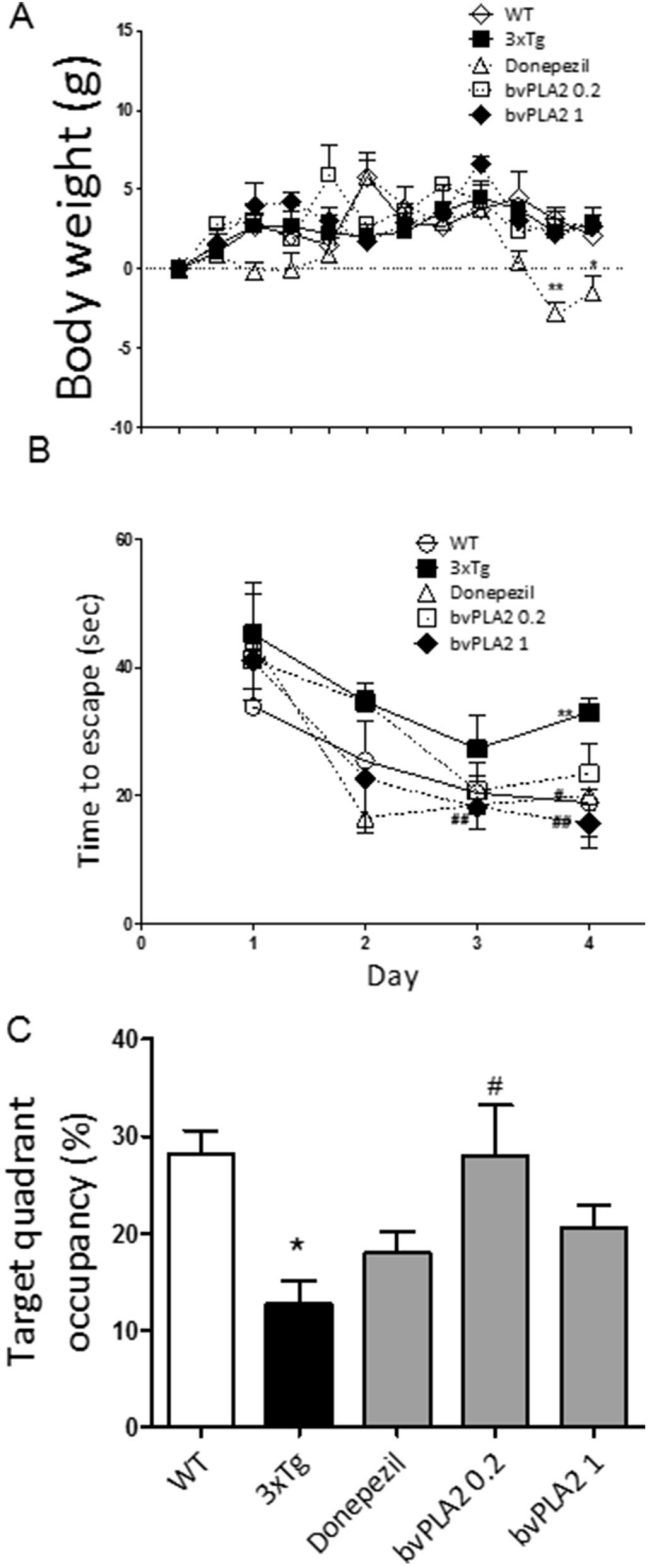
The effects bvPLA2 on the body weights and behaviors of the 3xTg mice. The body weight curves for the different groups of experimental animals were determined (**a**). *WT* PBS-treated wild type group, *3xTg* 3xTg group, *donepezil* donepezil-treated 3xTg group, *bvPLA2 0.2* bvPLA2 0.2 mg/kg-treated 3xTg group, *3xTg+bvPLA2* bvPLA2 1 mg/kg-treated 3xTg group. The latencies to escape onto the hidden platform in the Morris water maze. The task was performed for three trials per day over 4 days for the acquisition test (**b**). Retention performance was tested on fourth day. In the retention test, the mice received a probe trial in which the platform was removed from the pool (**c**). The values are presented as the means ± the SEMs. *P < 0.05, **P < 0.01, compared to the WT group; #*P* < 0.05, compared to the 3xTg group (repeated-measures ANOVA with and Tukey’s post hoc analyses)

The body weights of the wild type (WT), donepezil-treated, bvPLA2-treated, and vehicle-treated 3xTg AD mice were measured every week during the 3-month treatment (Fig. 1a). There were no obvious effects of bvPLA2-treatment (3 to 6 months of age) on the body weights compared to the vehicle-treated 3xTg AD mice. In contrast, the donepezil treatment group exhibited significantly decreased body weight compared to the vehicle-treated 3xTg AD group.

### bvPLA2 reverses the spatial learning deficits of 6-month-old 3xTg-AD mice

The latencies to escape onto the hidden platform during the acquisition trials of the water maze were recorded. The escape latencies differed between the groups when the results were averaged over all sessions. The latencies to find the hidden platform of the WT, donepezil, and bvPLA2 (0.2 and 1 mg/kg) groups were significantly shorter than those of the 3xTg AD group (Fig. 1b).

The total times spent in the quadrant that previously contained the platform were used to evaluate the spatial performances of the mice the during retention trials. The results of the retention tests on the fifth day are depicted in Fig. 1c. The bvPLA2-treated group (0.2 mg/kg) exhibited a significant increase (*P* < 0.05) in retention time compared to the 3xTg AD group (Fig. 1c).

### Effects of bvPLA2 on Aβ pathology in the 3xTg-AD mice

**Fig. 2:**
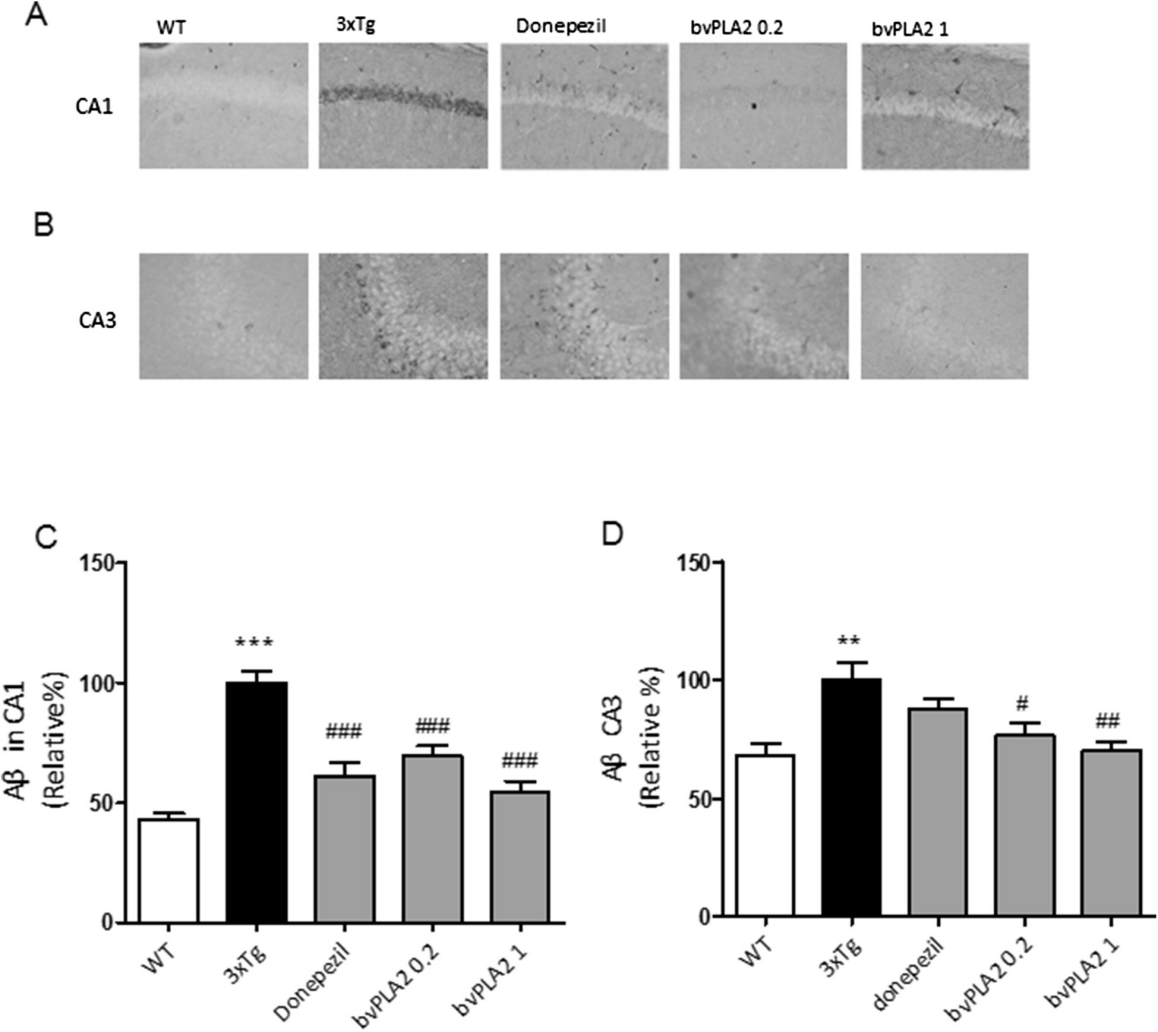
Effects of bvPLA2 on Aβ deposition in the hippocampi of 6-month-old 3xTg-AD mice. Aβ burdens were detected with immunohistochemical staining with anti-Aβ antibodies in the hippocampal regions of CA1 (**a**, **c**) and CA3 (**b**, **d**). The data are shown as the means ± the SEMs; **P < 0.01, ***P < 0.001 compared to the WT group; ^#^ *P* < 0.05, ^##^ *P* < 0.01, ^###^ *P* < 0.001, compared to the 3xTg group (repeated-measures ANOVA with Tukey’s post hoc analyses)

In 3xTg-AD mice, Aβ deposits are present in the hippocampus by 6 months of age [16, 17]. Quantifications of the Aβ burden revealed significantly increased burden in the hippocampal CA1 and CA3 regions of 3xTg AD group at the age of 6 months compared to the age-matched WT group (100 ± 4.72, *P* < 0.001, 100 ± 7.6, *P* < 0.001, respectively, Fig. 2c, d). Compared to the vehicle-treated 3xTg AD group, the Aβ burden in CA1 was dramatically decreased following bvPLA2 treatment (0.2 mg group 69.7 ± 5.84 %, *P* < 0.001 and 1 mg group 54.2 ± 4.79 %, *P* < 0.001, Fig. 2a, c). Similarly, donepezil treatment also decreased the Aβ burden in the CA1 region (60.9 ± 5.84, *P* < 0.001, Fig. 2). In contrast, only the CA3 regions of the bvPLA2-treated groups exhibited significant decreases in the amounts of Aβ burden (0.2 mg group 76.4 ± 5.42 %, *P* < 0.05 and 1 mg group 70.3 ± 3.72 %, *P* < 0.01, Fig. 2d), and there were no significant differences between the donepezil treatment group and the vehicle-treated 3xTg AD group.

### Changes in brain glucose metabolism

**Fig. 3:**
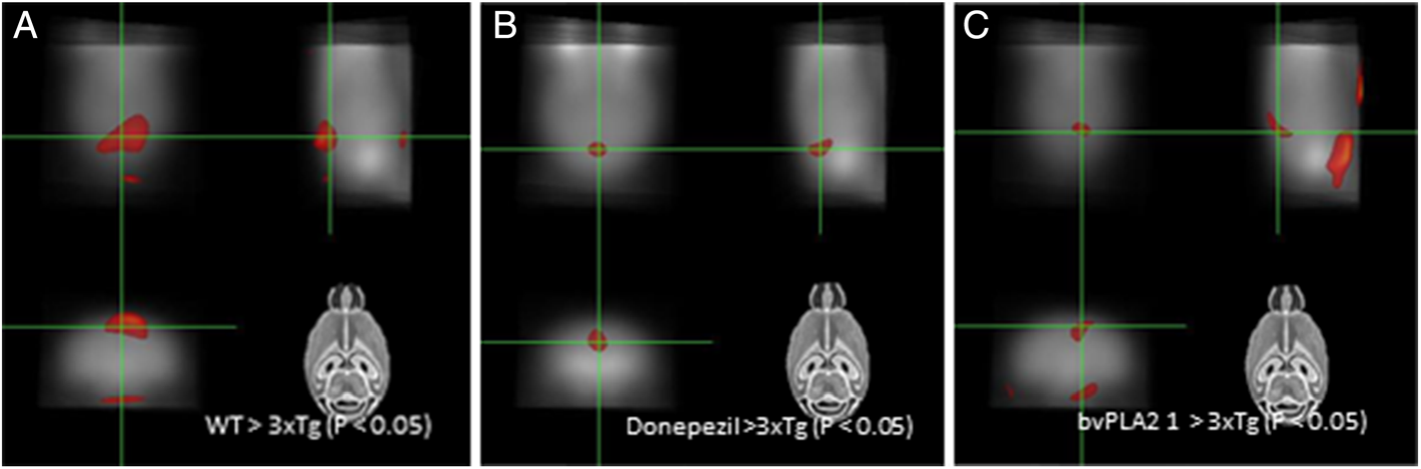
bvPLA2 increases glucose uptake in the brain. **a** Voxel-wise comparisons between the WT and 3xTg datasets. The brain regions in which regional FDG uptake in the WT were significantly greater than those in the 3xTg group (diencephalon). **b** Voxel-wise comparisons between the 3xTg and 3xTg+donepezil groups. Brain regions in which the regional FDG uptakes in the donepezil treatment group were significantly greater than those in the 3xTg group (cortex). **c** Voxel-wise comparisons between the 3xTg and 3xTg+bvPLA2 1 mg/kg groups. The brain regions in which the regional FDG uptakes in the bvPLA2 treatment group were significantly greater than those in the 3xTg group (diencephalon)

In vivo analyses of the brain glucose metabolism of live mice were performed using positron emission tomography. FDG-PET imaging scans indicated differences in the cerebral glucose metabolism rates from the hippocampi to the prefrontal cortices between the WT and 3xTg-AD groups (Fig. 3a), the 3xTg-AD and 3xTg-AD+donepezil groups (Fig. 3b), and the 3xTg-AD and 3xTg-AD+bvPLA2 groups (Fig. 3c). The SPM analysis revealed that the glucose metabolism in the diencephalon of the WT mice was significantly increased compared to that of the 3xTg-AD mice (*P* < 0.05). The glucose metabolism of the donepezil treatment group in the cortex was significantly increased compared to that of the 3xTg-AD group (*P* < 0.05). The glucose metabolism of the bvPLA2-treated group in the diencephalon was significantly increased compared to that of the 3xTg-AD group (*P* < 0.05).

### bvPLA2 reduces neuroinflammatory responses in the hippocampi of the 3xTg-AD mice

**Fig. 4:**
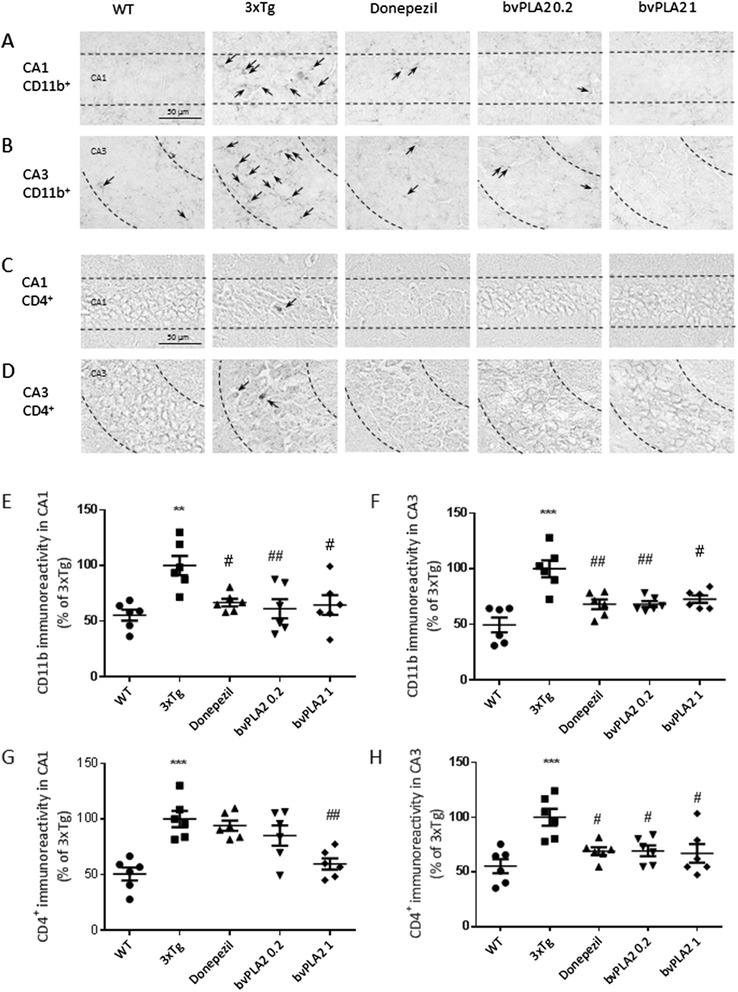
bvPLA2 reduces neuroinflammatory responses and the infiltration of CD4^+^ T cells into the hippocampi of 3xTg-AD mice. Treatment with bvPLA2 induced microglial deactivation in the hippocampal CA1 (**a**, **e**) and CA3 regions (**b**, **f**). Treatment with bvPLA2 induced reductions in CD4^+^ T cell infiltration in the hippocampal CA1 (**c**, **g**) and CA3 regions (**d**, **h**). The representative immunostained cells were indicated as *arrows* (**a**–**d**). The *error bars* indicate the SEMs. **P < 0.01, ***P < 0.001 compared to the WT groups; #*P* < 0.05, ##*P* < 0.01 compared to the 3xTg group (one-way ANOVA with Tukey’s post hoc analyses)

To determine whether the neuroprotective effects of bvPLA2 resulted from the inhibition of microglial activation in the hippocampus, immunostaining of brain sections anti-CD11b was performed (Fig. 4). In the WT group, only a few CD11b^+^ microglia/macrophages with resting morphologies (i.e., small cell bodies and thin processes) were observed in the hippocampi. In contrast, numerous CD11b^+^ microglia/macrophages with activated morphologies (i.e., larger cell bodies and thick processes) were apparent in the hippocampi of the 3xTg AD group. However, the majority of the CD11b^+^ microglia/macrophages in the bvPLA2-treated group had returned to a resting morphology in the hippocampi.

### bvPLA2 reduces the infiltration of CD4+ T cells into the hippocampi of 3xTg-AD mice

To determine whether bvPLA2 modulated the infiltration of CD4^+^ T cells into the hippocampi of the 3xTg-AD mice, we performed immunostaining of the hippocampi with an anti-CD4 antibody. The bvPLA2 treatment groups exhibited reduced numbers of infiltrating CD4^+^ T cells in the hippocampus compared to the 3xTg AD mice (Fig. 4).

### Neuroprotective effects of bvPLA2 are associated with the modulation of the peripheral immune tolerance of Tregs

**Fig. 5:**
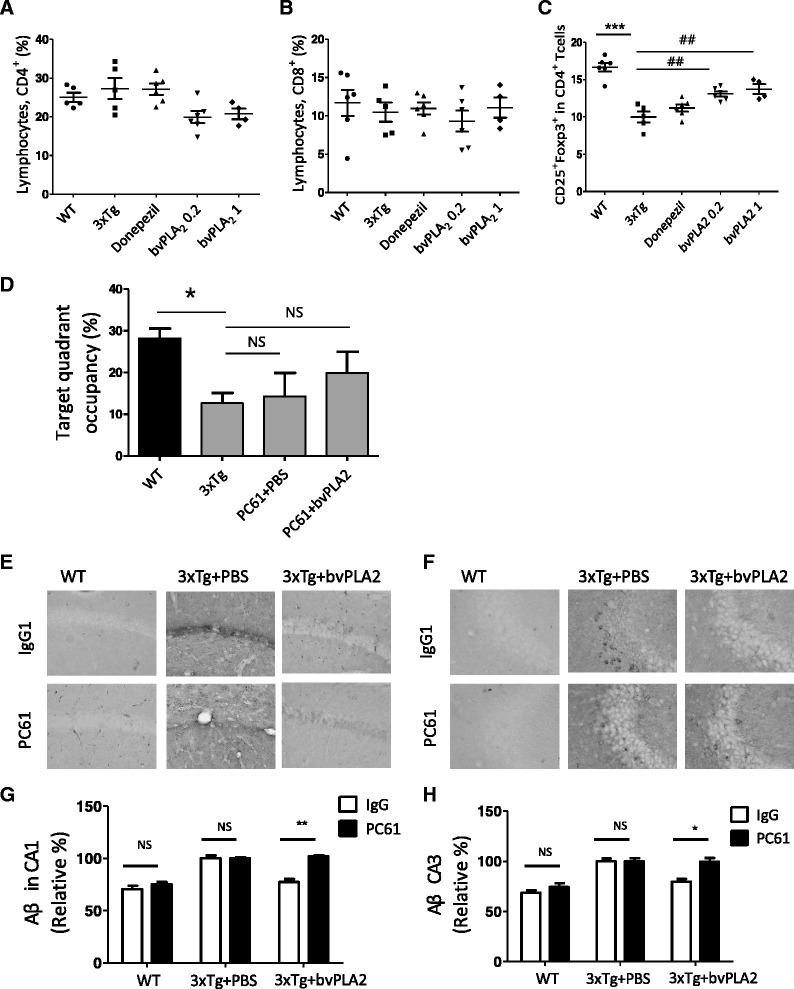
**a** The percentages of CD4^+^ were determined in the splenocytes. **b** The percentages of CD8^+^ were determined in the splenocytes. **c** The percentages of CD4^+^CD25^+^Foxp3^+^ Tregs were determined in the splenocytes of the *WT* PBS-treated wild type group, *3xTg* 3xTg group, *donepezil* donepezil-treated 3xTg group, *bvPLA2 0.2* bvPLA2 0.2 mg/kg-treated 3xTg group, *3xTg+bvPLA2* bvPLA2 1 mg/kg-treated 3xTg group. To deplete the Tregs, PC61 anti-CD25 mAb (1 mg/kg) was i.p. injected for 3 months once a week until they were 6 months old. The *error bars* represent the SEMs. ***P < 0.001 compared to the WT groups; ^##^ *P* < 0.01, ^###^ *P* < 0.001 compared to the 3xTg group (one-way ANOVA with Tukey’s post hoc analyses). **d** Retention performance was tested on fourth day. In the retention test, the mice received a probe trial in which the platform was removed from the pool. **e** The brain sections were prepared and immunostained with Aβ antibody to identify the Aβ deposits in CA1. **f** The Aβ in the hippocampal CA3 region as detected with immunohistochemical staining with using anti-Aβ antibody. **g** Measured depositions of Aβ in CA1. **h** Measured depositions of Aβ in CA3. The *error bars* represent the SEMs. *P < 0.05 and **P < 0.01 (two-way ANOVA with Tukey’s posttests). *IgG1* normal rat IgG1, *PC61* PC61 anti-CD25 monoclonal antibody

To ascertain whether the increase in Tregs caused by bvPLA2 treatment was directly involved in the Aβ neuroprotective actions, the mice received PC61 anti-CD25 mAb to deplete Tregs or normal anti-rat IgG1 as an isotype control. PC61 anti-CD25 mAb is generally used to deplete of Tregs, and this depletion is mediated by the antibody-dependent cellular-phagocytosis of FcγRIII^+^ macrophages in vivo [32]. Consistent with our previous data [33], the injection of the PC61 anti-CD25 mAb resulted in the depletion of over 90 % of the Foxp3^+^ cells in the CD4^+^-gated splenocytes (data not shown). The brain sections were immunostained with an anti-Aβ antibody. Aβ burden did not differ following Treg depletion in the 3xTg AD mice, and the neuroprotective effects of bvPLA2 were abolished in the Treg-depleted 3xTg AD mice (Fig. 5). Neither PC61 anti-CD25 mAb nor normal anti-rat IgG alone had any effect on Aβ burden in the hippocampus (Fig. 5). Next, we performed Morris water maze to measure cognition dysfunctions. The results demonstrated that there were no significant differences in the latency between bvPLA2 and PBS group after Treg depletion by PC61 anti-CD25 mAb injections (Fig. 5). These results strongly indicate that the neuroprotective effects of bvPLA2 were closely associated with Tregs in the 3xTg AD mice.

### Effects of bvPLA2 on CD4^+^ CD25^+^ Foxp3^+^ T cell populations

To determine whether bvPLA2 possessed immune tolerance-enhancing activity that was mediated by Treg induction, T cell sub-populations in splenocytes were analyzed in splenocytes. The percentages of CD4^+^ CD25^+^ Foxp3^+^ T cells in the splenocytes were reduced in the 3xTg AD group compared to the WT group, while CD4^+^, CD8^+^, and total lymphocytes were not altered. Treatment with bvPLA2 significantly increased the Treg population without altering the other lymphocytes, which suggests that bvPLA2 might suppress neuroinflammatory responses via Treg induction in 3xTg AD mice (Fig. 5).

## Discussion

AD is the most common cause of dementia, and this disease accounts for 60–80 % of all dementia [34]. The neuropathology of AD includes Aβ plaques and neurofibrillary tangles that are accompanied by neuroinflammation that is characterized by astrocytosis and microgliosis. Microglia that are attracted to Aβ deposits represent the primary cellular component that is associated with AD neuroinflammation [35]. In the present study, we confirmed the hypothesis that bvPLA2 improved cognitive functioning and reduced the accumulation of Aβ depositions in the brain. Moreover, we demonstrated that the neuroprotective effects of bvPLA2 were associated with the suppression of microglial activation and the reduction of the infiltration of CD4^+^ T cells in a 3xTg mouse model of AD. Furthermore, compared to donepezil, bvPLA2 was similarly or more effective in reducing the majority of the cognitive and neuroinflammatory responses. Several studies have suggested that inflammation has a vital role in the progression of AD because Aβ can activate microglia and thus initiate an inflammatory response [36]. Additionally, lymphocyte infiltration might amplify the inflammatory response and cause more neuronal death [37, 38]. Although there are no clear T cell responses in the brains of patients with Alzheimer’s disease, understanding T cell responses and adaptive immunity has become important for immunotherapy for Alzheimer’s disease [5].

Tregs are pivotal players in the regulation of tolerance because they dampen harmful autoimmune T cells and constrain inflammation [39, 40]. Recently, the modulation of Tregs has been proposed as a potential therapeutic approach for neuroinflammation-mediated disorders including AD [41].

AD was one of the first neurodegenerative diseases that were connected to neurotoxic microglial activation [42]. Activated microglia neurotoxicity has gained a strong foothold in the field of AD research and is a vital contributor to Aβ accumulation because activated microglia can secrete diverse inflammatory molecules, such as IL-1, IL-6, IFN-γ, TNF-α, and free radicals, which are that are all associated with Aβ cascade during the occurrence and progression of AD [43, 44]. Consistently, our results show that microglial activation was increased in the 3xTg-AD mice. Moreover, the microglial activation in 3xTg-AD mice was significantly decreased by bvPLA2 treatment.

CD4^+^ T cells are involved in various pathological CNS conditions. CD4^+^ T cell-mediated autoimmune responses are crucial in the pathogenesis of multiple sclerosis and, to some extent, AD [45, 46]. A number of reports further suggest that T cells are activated in AD patients and that these cells exist both in the periphery and as infiltrates in the brain [46]. Our results demonstrated that CD4^+^ T cell infiltration into the hippocampus was also dramatically reduced by bvPLA2 treatment.

Tregs comprise 5 to 10 % of the peripheral CD4^+^ T cells [47, 48]. Increasing evidence has shown that Tregs exert neuroprotective activities via the suppression of microglia; these activities have been verified in two independent experimental paradigms; i.e., antibody-mediated Treg depletion [49] and adoptive transfer of Tregs [50]. Although there are many evidences that Treg has beneficial roles in some neurodegenerative diseases, there are few studies about the roles of Treg in AD. However, Subramanian et al. showed that the consistently reduced expression of Foxp3 in spleens from both young and old 3xTg-AD mice compared with normal mice [51]. Treg decrease was reported in AD patients’ PBMC [52].

In our previous report, we showed that bvPLA2 treatment significantly increased the population of Treg cells via CD206 receptor on dendritic cells. bvPLA2 was directly bound to CD206, and the increase the population of Treg cells by bvPLA2 was abolished in CD206 KO mice [53]. In the present study, we demonstrated that bvPLA2 was not neuroprotective in Treg-depleted mice using anti-CD25 antibody injections. These data indicated the possible involvement of Tregs in the effects of bvPLA2 that decrease microglial responses and protect against the accumulation of Aβ in the 3xTg-AD mouse.

There are two ways of Treg depletion. One way is using depletion of regulatory T cells (DEREG) mouse expresses a fusion protein of the human diphtheria toxin (DT) receptor (hDTR) and enhanced green fluorescent protein (eGFP) under control of the Foxp3 promoter [54]. Lahl et al. reported that ablation of Foxp3+ T reg cells in newborn DEREG mice led to the development of scurfy-like symptoms with splenomegaly, lymphadenopathy, insulitis, and severe skin inflammation [55]. Depletion of FOXP3^+^ Treg cells in 8- to 10-week-old DEREG promotes hypercholesterolemia and atherosclerosis [54]. The other way of Treg depletion is using antibody targeting the interleukin-2 (IL-2) receptor α-chain (CD25) on the surface of T cells. CD25^+^ Treg depletion by PC61 monoclonal antibody induced organ-specific autoimmune diseases [56], [57]. It has known that the phenotypes of Foxp3^+^ Treg-depleted mice and CD25^+^ Treg-depleted mice are not exactly same. In our study, we could not observe any significantly different phenotypes from Treg-depleted mouse using PC61 antibody.

In recent years, several types of genetically modified mice have been generated as potential models for studying neuroinflammation [58], such as the APP/PS1-KO mice reported by Weitz et al. [58] and the TgSwDI mice reported by Goñi et al. [59]. In the present study, we chose a triple-transgenic mouse model of AD. The 3xTg-AD mouse was recently created and harbors three disease-relevant genetic alterations; i.e., a human presenilin M146V knock-in mutation (PS1M146V), a human amyloid precursor protein Swedish mutation (APPswe), and the human tauP301L mutation. These mice develop plaques and tangles in a spatially and temporally dependent manner that is similar to pathological hallmarks that are observed in the brains of AD-afflicted individuals [15, 60]. Most conspicuously, this animal model is the first that was developed to facilitate the study of inflammation in the context of amyloid pathology. Cognitive impairment is present in 2-month-old 3xTg-AD mice [61], and Aβ deposits in the hippocampus and cortex have been detected in 6-month-old 3xTg-AD mice [17], which contrasts with other genetic AD models. These findings indicate that the pathological features of the 3xTg-AD mice that mimic AD remain stable. Classic and well-known symptoms of AD include problems with spatial learning and the presence of memory deficits.

Many epidemiological and functional neuroimaging [62] studies have shown that the dysregulation of energy metabolism in the brain is a significant causative factor in the development of dementia. Positron emission tomography (PET) imaging is a new scanning technique in medical research. [F-18] FDG micro-PET scanning recently became available for visualizing brain activity in small rodents [63, 64]. The present study used PET analyses to demonstrate that the cerebral glucose metabolism of the bvPLA2 dataset was considerably increased in the brain compared to that of the 3xTg AD mice. Thus, an important point of our study is that, despite the limited spatial resolution of the micro-PET system, we were able to detect focal changes in the brains of the 3xTg-AD mice. Donepezil is able to ameliorate cognitive deficits, and comparison of this effect to our results leads us to suggest that the potency of bvPLA2 appears to be comparable to that of donepezil. Although donepezil is promising, it has serious side effects that might outweigh its benefits [13]. The results of others and our own have revealed that, unlike bvPLA2, donepezil consistently induces major side effects and weight loss [65].

## Conclusions

Treatment with bvPLA2 attenuated learning and memory deficits exerted anti-neuroinflammation effects in 3xTg-AD mice. Based on the results of the present study, we conclude that bvPLA2 has potential for use in the treatment of AD.

## Abbreviations

AChE: acetylcholinesterase
AD: Alzheimer’s disease
Aβ: amyloid beta
bvPLA2: bee venom phospholipase A2
MPTP: 1-methyl-4-phenyl-1,2,3,6-tetrahydropyridine
PET: positron emission tomography
PFA: paraformaldehyde
Tregs: regulatory T cells
WT: wild type
